# Further structural insights into the binding of complement factor H by complement regulator-acquiring surface protein 1 (CspA) of *Borrelia burgdorferi*

**DOI:** 10.1107/S1744309113012748

**Published:** 2013-05-23

**Authors:** Joseph J. E. Caesar, Reinhard Wallich, Peter Kraiczy, Peter F. Zipfel, Susan M. Lea

**Affiliations:** a Sir William Dunn School of Pathology, University of Oxford, South Parks Road, Oxford OX1 3RE, England; b Institute of Immunology, University of Heidelberg, Im Neuenheimer Feld 305, 69120 Heidelberg, Germany; c Institute of Medical Microbiology and Infection Control, Frankfurt University Hospital, Paul-Ehrlich-Strasse 40, 60596 Frankfurt, Germany; d Department of Infection Biology, Leibniz Institute for Natural Products Research and Infection Biology, Beutenbergstrasse 11a, 07745 Jena, Germany; e Friedrich Schiller University of Jena, 07737 Jena, Germany; f Oxford Martin School of Vaccine Design, Sir William Dunn School of Pathology, University of Oxford, South Parks Road, Oxford OX1 3RE, England

**Keywords:** complement, factor H, BbCRASP-1, CspA, *Borrelia burgdorferi*

## Abstract

*Borrelia burgdorferi* has evolved many mechanisms of evading the different immune systems across its range of reservoir hosts, including the capture and presentation of host complement regulators factor H and factor H-like protein-1 (FHL-1). Acquisition is mediated by a family of complement regulator-acquiring surface proteins (CRASPs), of which the atomic structure of CspA (BbCRASP-1) is known and shows the formation of a homodimeric species which is required for binding. Mutagenesis studies have mapped a putative factor H binding site to a cleft between the two subunits. Presented here is a new atomic structure of CspA which shows a degree of flexibility between the subunits which may be critical for factor H scavenging by increasing access to the binding interface and allows the possibility that the assembly can clamp around the bound complement regulators.

## Abstract 2

*B. burgdorferi* binds complement factor H using a dimeric surface protein, CspA (BbCRASP-1). Presented here is a new structure of CspA that suggests that there is a degree of flexibility between subunits which may have implications for complement regulator binding.

## 1. Introduction   

*Borrelia burgdorferi* is a Gram-negative spirochete and is the causative agent of the most commonly occurring vector-borne disease in Europe and North America, Lyme borreliosis (Barbour & Hayes, 1986 ▶; Steere, 1989 ▶; Centres for Disease Control and Prevention, 2007 ▶). Following transmission into the dermis during feeding of infected *Ixodes* ticks, the predominant indication of infection is a spontaneously resolving skin rash (erythema migrans) often accompanied by other symptoms including headache and fever (Steere, 1989 ▶; Stanek & Strle, 2003 ▶). If the infection is not immediately cleared by host immunity or antibiotic treatment, the spirochetes can spread to major organs within the host, causing a chronic multisystemic disorder (Steere, 1989 ▶).

*Borrelia* species have developed many strategies for evading the different immune systems across their range of reservoir hosts, including the capture and presentation of host complement regulators, a mechanism that has been developed by many pathogenic bacteria (Embers *et al.*, 2004 ▶; Lambris *et al.*, 2008 ▶; Zipfel *et al.*, 2007 ▶). Resistance of distinct *Borrelia* species towards the complement response upon exposure to human serum has been linked to the binding of the major alternative pathway regulators factor H and factor-H-like protein-1 (FHL-1) by a family of molecules termed complement regulator-acquiring surface proteins (CRASPs; Kraiczy, Skerka, Brade *et al.*, 2001 ▶; Kraiczy, Skerka, Kirschfink, Brade *et al.*, 2001 ▶; Kraiczy, Skerka, Kirschfink, Zipfel *et al.*, 2001 ▶; Stevenson *et al.*, 2002 ▶).

Factor H is a 155 kDa plasma protein consisting of 20 short consensus-repeat (SCR) domains. The four N-terminal domains possess decay-accelerating activity towards the alternative pathway C3 convertase and act as a cofactor for factor I-mediated cleavage of C3b (Pangburn *et al.*, 1977 ▶; Vik *et al.*, 1990 ▶; Whaley & Ruddy, 1976 ▶). The local concentration of factor H is increased on self-cell surfaces *via* interactions with glycosaminoglycans, characterized by heparin-binding sites found in domains 6 and 7 and 19 and 20 (Schmidt *et al.*, 2008 ▶; Prosser *et al.*, 2007 ▶). Bacteria have evolved surface protein glycosaminoglycan mimics that bind factor H in these regions in an escape mechanism that parallels that of host cells (Schneider *et al.*, 2009 ▶).

**Figure 1:**
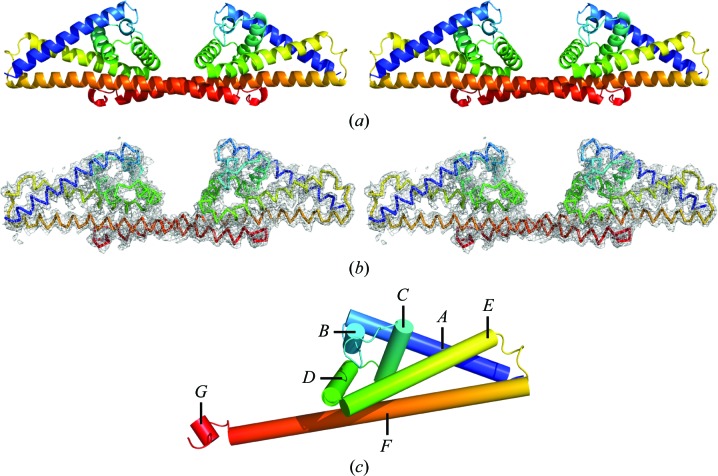
Structure of CspA (PDB entry 4bl4). (*a*) Stereoscopic views of the 4bl4 main chain shown in cartoon representation with and without the 2*F*_o_ − *F*_c_ electron-density map contoured at 1.5σ. Colouring runs from the N-terminus (blue) to the C-terminus (red). (*b*) Cylinder representation of the CspA subunit with helices numbered from the N-­terminus. This figure was generated using *PyMOL* (Schrödinger LLC).

CspA (also referred to as BbCRASP-1 or BBA68) is expressed on the surface of *B. burgdorferi* and binds factor H and FHL-1 in the region of domains 5–7 with an affinity measured in the range 10–30 n*M* (Kraiczy *et al.*, 2004 ▶). The atomic structure of CspA is known and consists of seven α-helices joined by short loops assembled in a ‘lollipop’-type arrangement (Cordes *et al.*, 2005 ▶). The crystal structure shows the formation of a homodimeric species mediated by inter­actions between helix *F* in both subunits. The dimeric structure possesses a cleft between the two subunits (Fig. 1 ▶) within which a putative factor H binding site has been proposed following *in vitro* mutagenesis studies (Cordes *et al.*, 2006 ▶; Kraiczy *et al.*, 2009 ▶). The same studies also highlighted the importance of the ten C-terminal residues forming helix *G*. Deletion of these residues destabilizes dimer formation, resulting in abolition of factor H binding. These C-­terminal residues bind in a tight pocket formed on the second subunit, which suggests that these residues are responsible for locking the dimer together.

Presented here is a new crystal structure of CspA showing a different conformation between the dimer subunits, demonstrating a degree of flexibility which has implications for the accessibility and conformation of the binding site. Despite flexibility in the dimer organization, the C-terminal lock structure is completely conserved, suggesting that these interactions underpin the assembly and therefore the biological activity of CspA.

## 2. Experimental   

### 2.1. Protein expression and purification   

A CspA construct encoding residues 70–250 was expressed and purified as described previously (Kraiczy *et al.*, 2004 ▶). A final size-exclusion gel-filtration step was performed using an S-200 16/60 column (GE Healthcare) equilibrated in 50 m*M* Tris, 150 m*M* NaCl pH 7.2.

### 2.2. Crystallization and data processing   

Crystals were grown at 294 K using vapour diffusion in 400 nl sitting drops produced by an Oryx Nano crystallization robot (Douglas Instruments, UK). Each drop consisted of a 1:1 ratio of mother liquor (18% PEG 8000, 5 m*M* zinc acetate, 100 m*M* sodium cacodylate pH 6.5) and protein solution (*A*_280_ = 5.20). Crystals were cryoprotected using 15% ethylene glycol and data were collected on beamline ID23-2 at the ESRF, Grenoble, France. Data were processed with the *xia*2 (Winter, 2010 ▶) data-processing suite, which uses the programs *XDS* (Kabsch, 2010 ▶) and *SCALA* (Evans, 2006 ▶) (Table 1 ▶).

### 2.3. Structure determination and refinement   

**Figure 2:**
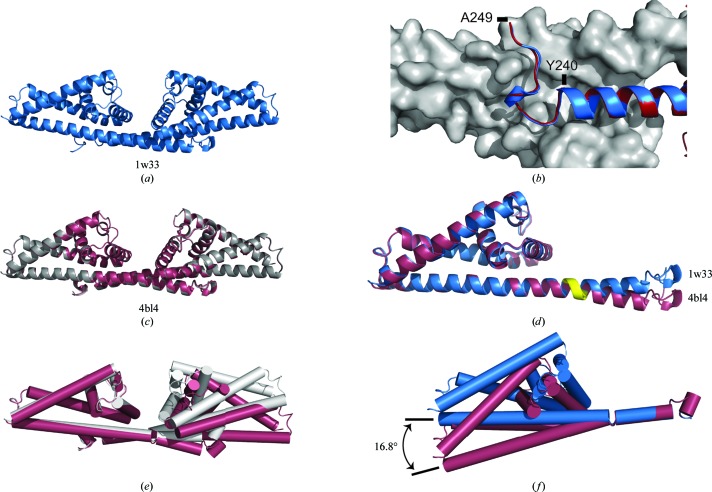
Comparison between PDB entries 4bl4 and 1w33. (*a*) Cartoon representation of the structure of the CspA dimer found in PDB entry 1w33. (*b*) Superposition (r.m.s.d. = 0.138 Å) of C-terminal residues 230–250 from chain *A* in 4bl4 (red) and 1w33 (blue) shown as a cartoon against chain *B* rendered as a surface (grey). (*c)* Cartoon representation of the structures of both copies of the CspA dimer found in the asymmetric unit of PDB entry 4bl4. The dimer formed by chains *C* and *D* (grey) has been superimposed on that formed by chains *A* and *B* (red) using secondary-structure matching (Krissinel & Henrick, 2007 ▶), with an r.m.s.d. of 0.264 Å. (*d*) Subunits from 1w33 (blue) and 4bl4 (red) superposed, showing the deflection of helix *F* between residues 225 and 227 (shown in yellow). (*e*) Overlay of the dimer assemblies found in 1w33 and 4bl4, showing the difference in the intermonomer angle. Chains *A* from 1w33 (grey) and 4bl4 (red) were superposed using secondary-structure matching over residues 70–220. The average r.m.s.d. of superposition of each chain in 1w33 onto each chain in 4bl4 over this residue range is 0.539 Å. (*f*) Superposition of C-terminal residues from chain *A* of 1w33 and 4bl4, showing an average increase in intermonomer angle of 16.8° averaged over both copies of the dimer in the 4bl4 asymmetric unit (the individual angles for the *AB* and *CD* dimers are 16.96 and 16.66°, respectively). This figure was generated using *PyMOL* (Schrödinger LLC).

The structure was solved in space group *C*2 by molecular replace­ment using the existing CspA structure (PDB entry 1w33 chain *A*; Cordes *et al.*, 2005 ▶) and *Phaser* (McCoy *et al.*, 2007 ▶) from the *CCP*4 suite (Winn *et al.*, 2011 ▶). The solution was refined iteratively using *Coot* (Emsley *et al.*, 2010 ▶) and *autoBUSTER* (Blanc *et al.*, 2004 ▶; Bricogne *et al.*, 2011 ▶), using local secondary-structure target restraints (Smart *et al.*, 2008 ▶) to minimize the risk of overfitting. *B* factors were not refined and were set to those of the initial model. Refinement statistics may be viewed in Table 2 ▶ and stereo images of the protein main chain alongside representative electron density are presented in Fig. 2 ▶. The refined structure was validated using *MolProbity* (Chen *et al.*, 2010 ▶), which gave a score of 1.3 and placed it in the 100th percentile of structures in the 3.25–4.31 Å resolution range. The final coordinates were deposited in the PDB with accession code 4bl4.

### 2.4. Structural analyses   

The residues involved in the bending of helix *F* were highlighted using *DynDom* (Hayward & Berendsen, 1998 ▶). Measurement of the difference in inter-domain angles between PDB entries 4bl4 and 1w33 was performed using secondary-structure matching over residues 70–­220 with *LSQKAB* (Kabsch, 1976 ▶).

## 3. Results and discussion   

**Figure 3:**
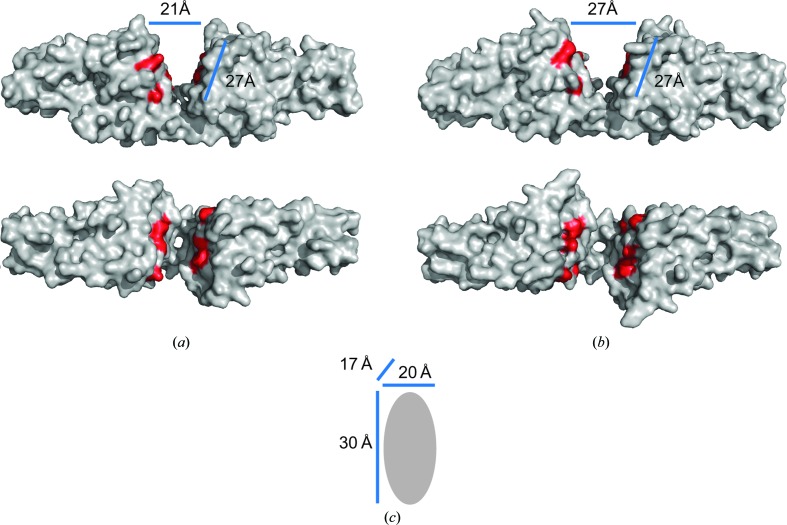
Implications of the factor H/FHL-1 binding site. (*a*, *b*) Surface representations of dimers from PDB entries 1w33 and 4bl4, respectively, highlighting residues Lys136, Lys141, Lys143, Glu144 and Glu47 which have been shown to be involved in factor H/FHL-1 binding (Kraiczy *et al.*, 2009 ▶). Differences in the dimensions of the cleft between the subunits are also shown. (*c*) Typical dimensions of a single SCR domain. This figure was generated using *PyMOL* (Schrödinger LLC).

The structure of CspA from *B. burgdorferi* has been redetermined to 4.1 Å resolution in a new crystal form, showing that the bacterial protein exists in a dimeric form which is highly similar to that observed in the previous crystal structure (Figs. 1 ▶ and 2 ▶). Comparing the conformation of both copies of the dimer in the asymmetric unit with that in PDB entry 1w33 shows that the angle between the subunits has increased by an average of 16.8° (Fig. 2 ▶). This finding suggests that the assembly possesses a degree of flexibility between the subunits that results in a widening of the cleft compared with the original structure and may result in increased access to the putative binding site suggested by *in vitro* mutagenesis data (Fig. 3 ▶). It may also be possible for the CspA dimer to ‘clamp’ around a factor H or FHL-1 molecule bound within the cleft.

The differences between the two CspA structures are likely to arise from the different crystal packings. However, these structures illustrate that flexibility between the subunits is mediated by distortion of helix *F* between residues 225 and 227. This leaves the structure of the ten C-terminal residues (240–250) unaffected (Fig. 2 ▶), supporting earlier evidence that these residues are required for the assembly of a stable dimer (Cordes *et al.*, 2006 ▶). Further weight is also added to the hypothesis that the dimeric species is essential for the function of CspA as the ‘lock’ to the second subunit is unaffected.

Our findings suggest that the CspA dimer has a degree of flexibility which could allow increased accessibility to the factor H/FHL-1 binding site and may enable it to ‘clamp’ around a bound SCR domain. Flexibility also allows alteration of the binding-site confirmation which could enable binding to different complement regulators, perhaps providing a key role of CspA in enabling *B. burgdorferi* to evade complement-mediated killing.

## Supplementary Material

PDB reference: CspA, 4bl4

## Figures and Tables

**Table 1:** Data-collection and processing statistics Values in parentheses are for the highest resolution shell.

Diffraction source	ID23-2, ESRF
Detector	MAR 225
Temperature (K)	120
Space group	*C*2
Unit-cell parameters (, )	*a* = 115.27, *b* = 44.16, *c* = 186.54, = 90.00, = 90.69, = 90.00
No. of molecules in unit cell *Z*	16
Matthews coefficient *V*_M_ (^3^Da^1^)	2.75
Solvent content (%)	53.3
Resolution ()	93.264.06 (4.164.06)
*R*_merge_	0.194 (0.596)
*I*/(*I*)	5.7 (2.7)
Completeness (%)	95.9 (98.6)
Multiplicity	3.7 (3.7)
Data-processing software	*xia*2
Phasing method	Molecular replacement
Search model	1w33 chain *A*
Solution software	*Phaser*

**Table 2:** Structure refinement and model validation Values in parentheses are for the highest resolution shell.

Refinement software	*autoBUSTER*
Refinement on	*F*
Resolution ()	93.264.06 (4.544.06)
No. of reflections	7626 (1953)
No. of reflections for *R*_free_	747 (207)
*R*_work_/*R*_free_	0.2621/0.2707 (0.2675/0.2701)
No. of atoms	
Protein	6004
Ligand/ion	0
Water	0
R.m.s. deviations	
Bond lengths ()	0.009
Bond angles ()	1.10
Ramachandran plot analysis, residues in	
Most favoured regions (%)	97.3
Disallowed regions (%)	0.00
